# Etymologia: Coronavirus

**DOI:** 10.3201/eid2605.ET2605

**Published:** 2020-05

**Authors:** Ronnie Henry

**Affiliations:** Centers for Disease Control and Prevention, Atlanta, Georgia, USA

**Keywords:** coronavirus, viruses, avian infectious bronchitis virus, severe acute respiratory syndrome, SARS, severe acute respiratory syndrome coronavirus, Middle East respiratory syndrome, MERS, Middle East respiratory syndrome coronavirus, 2019 novel coronavirus disease, COVID-19, severe acute respiratory syndrome coronavirus 2, SARS-CoV 2, respiratory infections, zoonoses

## Coronavirus [kǝ-roʹnǝ-viʺrus]

The first coronavirus, avian infectious bronchitis virus, was discovered in 1937 by Fred Beaudette and Charles Hudson. In 1967, June Almeida and David Tyrrell performed electron microscopy on specimens from cultures of viruses known to cause colds in humans and identified particles that resembled avian infectious bronchitis virus. Almeida coined the term “coronavirus,” from the Latin *corona* (“crown”), because the glycoprotein spikes of these viruses created an image similar to a solar corona (Figure).

**Figure:**
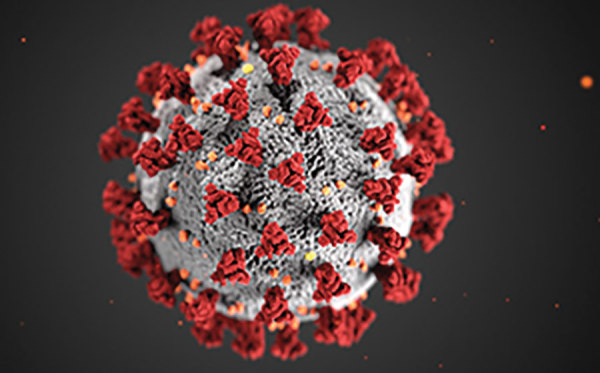
Illustration reveals the ultrastructural morphology exhibited by coronaviruses. Note the spikes that adorn the outer surface of the virus, which impart the look of a corona surrounding the virion, when viewed electron microscopically. Photo: CDC/ Alissa Eckert, MS; Dan Higgins, MAMS

Strains that infect humans generally cause mild symptoms. However, more recently, animal coronaviruses have caused outbreaks of severe respiratory disease in humans, including severe acute respiratory syndrome (SARS), Middle East respiratory syndrome (MERS), and coronavirus disease (COVID-19).
